# Matrix Stiffness Modulates Mechanical Interactions and Promotes Contact between Motile Cells

**DOI:** 10.3390/biomedicines9040428

**Published:** 2021-04-15

**Authors:** Subhaya Bose, Kinjal Dasbiswas, Arvind Gopinath

**Affiliations:** 1 Department of Physics, University of California Merced, Merced, CA 95343, USA; sbose@ucmerced.edu (S.B.); kdasbiswas@ucmerced.edu (K.D.); 2 Department of Bioengineering, University of California Merced, Merced, CA 95343, USA

**Keywords:** cell motility, elasticity, durotaxis

## Abstract

The mechanical micro-environment of cells and tissues influences key aspects of cell structure and function, including cell motility. For proper tissue development, cells need to migrate, interact, and form contacts. Cells are known to exert contractile forces on underlying soft substrates and sense deformations in them. Here, we propose and analyze a minimal biophysical model for cell migration and long-range cell–cell interactions through mutual mechanical deformations of the substrate. We compute key metrics of cell motile behavior, such as the number of cell-cell contacts over a given time, the dispersion of cell trajectories, and the probability of permanent cell contact, and analyze how these depend on a cell motility parameter and substrate stiffness. Our results elucidate how cells may sense each other mechanically and generate coordinated movements and provide an extensible framework to further address both mechanical and short-range biophysical interactions.

## 1. Introduction

Many eukaryotic cells move by crawling, which is by adhering to and exerting mechanical stresses and local forces on their extracellular matrix (ECM) that they then actively deform (see, for instance, [1,2,3,4] and the references therein). For these motile cells, which include fibroblasts, endothelial, and muscle cells, among others, the mechanical properties such as viscosity and elasticity of the environment on or in which the cell moves is known to play crucial roles in determining cell and tissue structure and function [5,6]. The biological relevance of mechanical signaling between cell and substrate or between two or more cells, as opposed to more extensively studied inter-cellular chemical signaling, was dramatically illustrated in [7], where it was shown that the lineage specification of stem cells can be directed by varying only the substrate stiffness. Much recent progress has been made since this pioneering work in understanding the role of mechanical forces in biology, and how biomolecules, especially those in the cellular force-generating and surface-sensing systems and signalling networks, respond to mechanical forces through the process of mechanotransduction [5,8,9,10,11,12,13,14,15,16].

Existing approaches to modeling collective cell motility focus on direct (steric and adhesive) cell–cell interactions or focus at the single cell level on cell-substrate interactions [2]. The latter deals with details of focal adhesions that are crucial in generating traction stresses in both adherent and motile cells [17]. Experiments surprisingly and strongly indicate that cells cultured on soft, elastic, bio-compatible substrates can respond to each other even when not in direct contact [3,4]. Such non-contact and long-range cell–cell interactions often arise when the cells are cultured. A common method is to culture cells on the surface of synthetic hydrogels such as polyacrylamide, a linearly elastic polymer. In this instance, cells that are spatially separated may still sense each other through mutual and active deformations of the gel by the cells. These mechanically derived non-contact cell–cell interactions are even more relevant and they act over longer ranges in the biological extracellular matrix (ECM) comprising collagen or fibrin, where cells can interact by remodeling and reorienting the fibers in the ECM [18,19,20]. Even without such cell–matrix feedback, the presence of matrix or substrate deformations has recently been shown to guide the migration of other cells without requiring chemotactic cues [21].

In this context, it is useful to compare non-contact and long range mechanical signalling, as analyzed by these studies, to cell–cell interactions that are not mechanical; specifically, chemical signalling, such as in immune cell interactions (for instance, see the review by [22] and references therein), or other forms of interactions, such as haptotaxis [23,24,25,26,27,28], cell stimulation by activating or secretory molecules, such as in inflammation and thromboses [29,30]. Mechanical non-local interactions between cells offer advantages as compared to chemical means in terms of the rate at which cells can communicate. Mechanical signaling and mechanosensing of neighbouring cells is typically faster and longer-ranged than chemical signaling and concentration field based interactions that are limited by slow diffusion rates. This is because mechanical interactions propagate near instantaneously [31]. This crucially allows cells to not just sense each other, but also synchronize their behaviour quickly at time scales faster than that seen in diffusive processes. For instance, substrate deformation-mediated long-range interactions have been clearly demonstrated in heart muscle cells that synchronize their beating without direct contact [32,33], as well as at a subcellular level between myofibrils within a single heart muscle cell [34]. Cell communications via the sensing of substrate or matrix deformation are particularly important in sparse, non-confluent cell cultures, or tissue that occur in a number of biologically relevant situations. Apart from beating cardiomyocytes, examples of such situations include wound healing involving fibroblasts [35], sprouting blood vessels comprising endothelial cells [36], and the migration of mesenchymal cells in zebrafish embryo before the formation of confluent epithelial tissue [37]. In all of these cases, cells are not in direct contact, but they exert traction forces on the surrounding mechanical medium and concomitantly sense deformations that are caused by nearby cells. Therefore, such interactions crucially depend on the stiffness of the substrate, and they can be probed by experiments that vary the stiffness of the hydrogel substrate on which the cells are cultured [5,38]. These aspects influence not only motility response at the single cell level, but they also strongly impact collective behavior, including directed motility and subsequent spatial self-organization.

While substrate-mediated cell–cell elastic interactions have been considered for the organization of adherent cells in a variety of mechanobiological contexts [39,40] (the physical basis of such modeling is reviewed in [41]), their effect on collective cell motility, which, in principle, is always present, have not been carefully modeled. Here, we present a simple biophysical agent-based model and computational results that focus on how substrate-mediated mechanical communication allows two cells to sense each other and impacts their collective and relative motility. The focus on mechanical interactions allows us to clearly explore the role of mechanical signaling. By itself, this minimal model can be used to describe cell movements in tissue culture experiments and guide applications that involve varying the mechanical properties of the cellular microenvironment.

Chemical signaling between cells and attendant biochemistry and kinetics is undeniably important in a biological context, and it is expected to act in concert with mechanical substrate mediated interactions between cells. However, our model provides a foundation for the study of more general cell interactions that include both mechanical and chemical signalling, and generally short-range near-contact and long-range interaction modalities. For instance, we have used a similar methodology to study short range interactions for prokaryotic cells, such as bacterial swarms moving on substrates and responding to chemical cues [42]. Our approach also serves as a starting point for studies of mechanical substrate based interactions in multi-cellular systems, such as growing tissue and confluent sheets.

## 2. Experimental Observations Motivate Model for Cell Elastic Interactions

**Figure 1:**
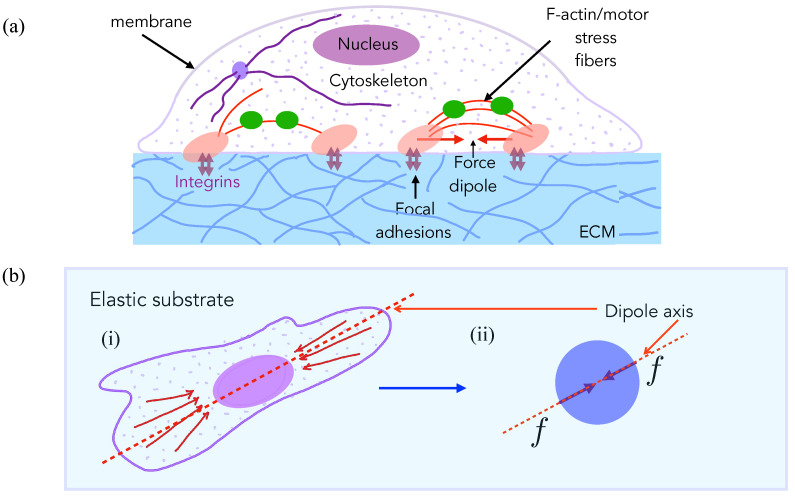
Traction forces that are exerted by motile cells on soft substrates can be modeled as force dipoles. (**a**) Schematic of an animal cell, e.g., an isolated fibroblast in culture [17] adhered to a compliant substrate through focal adhesions comprising of integrins and a host of other mechanosensitive adhesion proteins. Mechanical forces are actively generated by molecular motors of the myosin-II family that put the actin cytoskeleton under tension. Stress fibers are bundles of crosslinked actin filaments with a periodic (sarcomeric) organization of myosin [44] that often span the length of the cell and are anchored at the focal adhesions to the extracellular substrate or matrix (ECM). The contractile forces are transmitted at these sites from the stress fibers to the underlying substrate, which can be strongly deformed if soft. Such deformations are long range (they extend up to a few cell lengths away from the cell) and they can be measured by Traction Force Microscopy (TMF). This is a common index of cell–substrate mechanical interactions. (**b**) (i) A simplified top view of the same cell showing the alignment of the stress fibers and, therefore, of the contractile forces that are generated by them. In order to model the effects of the cell on the substrate, we use classical linear elasticity theory with the stress distributions effectively modeled as a contractile force dipole, a pair of equal and opposite forces separated by some distance acting along the dipole axis (marked). (ii) This leads to a very simplified mechanical model of the cell in terms as a contractile force dipole that is exerted on the substrate along its average axis of orientation defined by the alignment of its stress fibers.

**Figure 2:**
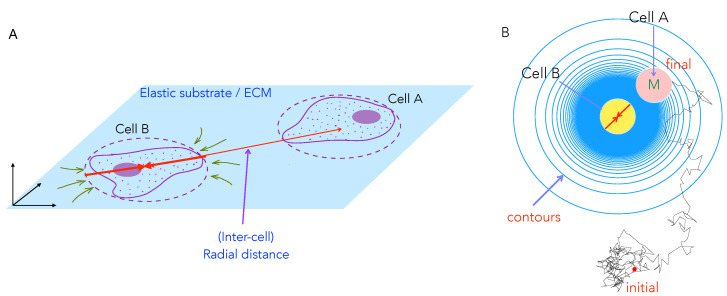
Schematic of the cell–cell mechanical interactions model: (**A**) Two cells A and B cultured on the surface of thick elastic substrate can sense each other and interact at long range (when the inter-cell distance *r* is longer than typical cell sizes, depicted here by dashed red circles) through mechanical deformations of the underlying substrate; here the contractile stresses set up in the substrate yield deformations as indicated by green arrows. The cells are restricted to move on the surface of the substrate. (**B**) We study with our computational model how a motile cell (M, Cell A, pink) moves in the presence of a fixed central cell (Cell B, yellow). This two cell system on a substrate (schematic shown as a top view) also mimics scenarios where a motile cell may encounter an elastic impurity or obstacle on the medium. Shown in blue circles are contours of constant elastic potential (in simplified form) that determine the inter-cell elastic force that is experienced by the motile cell B as a result of the elastic deformations of the medium by both cells A and B. Also shown (in black) is a representative simulated trajectory of the motile cell which starts outside the area of influence of the stationary cell.

Many eukaryotic cells use contractile localized forces that are generated by their actomyosin cytoskeleton to adhere to and move on their substrates (Figure 1a). Such traction forces typically cause measurable deformations in the underlying substrates in cell culture experiment [17], and they have a spatially dipolar pattern [43]. A cell typically acts as a force dipole exerting—a pair of equal and opposite forces—on the elastic medium (Figure 1b). The dipole arrangement arises since no external forces are present on the system (cell + substrate), and the cell, therefore, moves on its own accord by exploiting the resisting forces that are exerted by the substrate on the cell. The net effect of traction stresses is to contract or pull in the elastic material comprising the substrate towards the cell (Figure 2A).

Here, to focus our discussion, we use experiments on endothelial cells to build the elastic model. In a seminal study, [3] studied bovine aortic endothelial cells (BAECs) cells that were cultured on polyacrylamide based hydrogel substrates of varying stiffness. Endothelial cell migration and traction stresses were directly measured in this study. The number of cell extensions sent out toward an adjacent cell that result in cell–cell contact was also counted for a period of 6 h as a function of substrate compliance. To investigate the influence of substrate compliance on cell–cell interactions, pairs of cells on varying compliance gels, ranging from very soft (Young’s modulus, E = 500 Pa) to very stiff (E = 33,000 Pa), were examined. Three qualitatively different behaviors were observed. On the softest gels (500 Pa), cells were seen to touch and remain in contact for the duration of the experiment. Once in contact, the cells were seen to extend additional pseudopodia toward the adjacent cell, but the cells generally did not become significantly spatially separated. Cells on intermediate compliances studied (E = 2500 and 5500 Pa) were observed to contact, separate and retouch repeatedly. Once the cells contacted, they were also observed to generally not migrate significantly far from each other, unless the two-cell interaction was disturbed by a third cell. On the stiffest gels (33,000 Pa), the cells contacted and migrated away from each other, without the same repeated contact behavior that was observed for cells on intermediate compliance substrates. Thus, cells made stable contacts on very soft gels (Elastic modulus, 500 Pa), whereas they made repeated contacts and withdrawals on substrates of intermediate compliance (Elastic modulus, 2500–5500 Pa). The tracking of the collective migration of two-cells also showed a strong response to substrate stiffness. Specifically, pairs of endothelial cells could display hindered migration when compared to individual cells, strongly implying that these cells sensed each other through the matrix. Pairs of cells on softer gels also showed reduced collective migration in comparison to isolated cells.

Motivated by this experiment, here we model the motility characteristics of a two cell system (the schematic in Figure 2A) that captures how elastic deformations induced in the substrate allow cells to respond to each other. Our aim is the prediction of signatures of this interaction that are the most relevant to biological processes involving cell motility. Specifically, as in experiments, we seek to understand the effects of substrate stiffness on cell migration speeds, dispersivity, and on the frequency and manner of binary cell contacts. Once mechanical far-field interactions between cells can be modeled, we can, in further studies, consider additional near-field or contact chemical and bio-chemical interactions.

We consider a pair of cells that each adhere to, and exert stresses on, the underlying substrate, thereby deforming it, as shown in Figure 2A. As mentioned above, adherent and motile cells generate contractile stresses on the substrate via focal adhesions. Focal adhesions comprise of integrins and a host of other mechanosensitive adhesion proteins with contractile forces actively generated by actin-myosin aggregates that form stress fibers—bundles of cross-linked actin filaments that are anchored at the focal adhesions. The contractile forces are transmitted at these sites from the stress fibers to the underlying substrate. While the cell by itself is a soft substrate, studies have indicated that stiffness in the vicinity of the focal adhesion may be regulated and controlled so as to achieve locally large stiffness values in cellular domains involved in the formation of focal adhesions. Therefore, as a first approximation, we treat substrate elasticity as being the controlling parameter and treat the effective (actively maintained) stress fiber stiffness as much larger than the substrate stiffness. Next, the contractility of each cell is minimally described by a physical model of force dipoles –a pair of equal and opposite forces exerted on the substrate, and is thus a tensorial quantity [39]. Such modeling is inspired by the theory of deformations that are induced by inclusions in materials [45]. Unlike passive material inclusions, cells can actively regulate their force production in response to external mechano-chemical cues from the substrate, including the presence of other cells. Such complicating feedback effects in cell–cell interactions have also been theoretically considered [46,47], but we ignore these for simplicity here, and we treat contractility as an intrinsic cell property that is independent of underlying substrate matrix strain and stiffness.

We start with a model that mimics experimental setups and considers two cell interactionsm, as illustrated in Figure 2A. To simplify our study, we assume that one of the cells is motile (Cell A) and the other is stationary (Cell B). The stationary cell B is nonetheless *alive* in that it still deforms the substrate. The resulting deformation field, or equivalently the substrate mediated elastic potential, is sensed by the other, distant, motile cell A. The interaction potential between the cells, in turn, creates a mechanical force on the motile cell A. For polarized and elongated cells, the deformations have a dipolar spatial pattern (as described in Appendix A). However, here we consider a simplified scenario that is valid when cells reorient very fast in the time for them to translate and migrate (Appendix A, *§*3). This implies that the directions of the dipole axis of both cell A and of cell B fluctuate rapidly. This implies that cell A moves and feels an effectively isotropic, attractive interaction potential that decays with distance as $∼1/r^{3}$ (iso-surfaces shown as blue circles in Figure 2B).

Polarized cells may persistently propel themselves along their body axis; here, we consider cells that are unbiased and, thus, act in the absence of any orienting chemical field or signal and, therefore, randomly extend their pseudopodia in different directions. This response, together with thermal noise, is the origin of the diffusive behaviour observed as the cell moves. Such meandering cell trajectories can be characterized by a diffusion coefficient; similar features hold for cell-pairs, in which case one may evaluate the dispersion or migration coefficients. In reference to Figure 2B, as the motile cell A moves, it is additionally acted upon by an elastic interaction force that arises due to its interaction (via the substrate) with the stationary cell B. The motion of the motile cell (A) may then be described while using the Langevin equation. While such an approach has been previously proposed and validated with experiments on elastically coupled motile active particles, such as swarming bacteria [42,48], it has not been previously studied in conjunction with cell–cell dynamics on elastic substrates.

We note that the model can be easily generalized (as derived in Appendix A) to describe a pair of motile cells, since the interactions are pairwise and reciprocal. The interaction potential is not isotropic and it depends on both the inter-cell distance as well as on the instantaneous alignment of the cells’ dipole axes. Thus, the force on each cell (related to the gradient of the potential) depends on not just the relative positions of the cells, but additionally on the direction of the contractile dipoles that are exerted by cells A and B. Truly spherical dipoles that are embedded in an elastic medium do not interact mechanically [45], unless cell-substrate feedback effects occur [47]. Furthermore, cell–cell interactions in a fibrous, nonlinear elastic medium can be longer ranged [49], and they have a power law character, $∼1/r^{\alpha }$, where α<3 [50]. The interaction of disk-like cells on top of a thick substrate (semi-infinite geometry) is also more complicated [51]. We choose the isotropic, attractive $\;1/r^{3}$ potential as the simplest attractive interaction with the same distance dependence as the dipolar interaction, with the objective of testing how such a potential can affect cell motility. Motivating future work, we show how the conclusions from the simpler potential remain qualitatively valid, even as specifics of cell trajectories change when the more general dipolar potential is used. This model highlighted in this work, although very simplified in both its description of cell contractility and motility, can thus capture key aspects of motility and contact formation, as we now describe.

## 3. Materials and Methods

### 3.1. Model for Two-Cell Interactions

An agent-based stochastic model is the model that is used to analyze the two-cell system. We start with the stochastic Langevin equation for the dynamics of the moving cell *A* in the presence of a second cell *B* fixed at the origin, as illustrated in Figure 1a. Details of the model and the simplifications involved may be found in Appendix A. Starting from the more general model where both cells A and B can move, we now fix cell B and thus set $r^{B}=0$. In other words, we choose the center of cell B to be the origin from which the position of cell A and its distance relative to B is measured. Writing $r=r^{B}-r^{A}$, we write the equation for r(t), where *t* is the time,

(1) $$\frac{dr}{dt}=-\mu_{T}\frac{\partial W}{\partial r}+\sqrt{2D_{\mathrm{eff}}}\;\;\eta \left(t\right)$$

where $D_{\mathrm{eff}}$ is the effective translational diffusivity quantifying the random motion of the moving cell in the absence of the fixed cell, and η is a random white noise term whose components satisfy

$$\langle \eta_{i}\left(t\right)\eta_{j}\left(t^{\prime }\right)\rangle =\delta \left(t-t^{\prime }\right)\delta_{ij}.$$

Note that η—the active noise term—has units of $t^{-1/2}$. The mobility $\mu_{T}$ in Equation (1) quantifies the effective friction from the medium and it is inversely proportional to the cell size σ and inversely proportional to the viscosity at the surface. Here, it is assumed that the cells moving on a wet surface and that the fluid nature of the surface provides a viscous resistance opposing cell motion.

The two-cell potential *W* derives from the elastic interactions communicated via the linear deformation of the substrate (Appendix A, Equation (A5)), and it is given by,

(2) $$\begin{array}{ccc} W & = & \frac{1}{2}k{\left(\sigma -r\right)}^{2},\;\;\;\mathrm{when}\;\;\;0\leq r\leq \sigma ,\;\;\;\mathrm{and}\;\;\; \end{array}$$

(3) $$\begin{array}{ccc} & = & -\frac{P^{2}}{E}\frac{\phi (\nu )}{r^{3}},\;\;\;\;\;\mathrm{when}\;\;\;r>\sigma . \end{array}$$

Numerical solutions to Equation (1) are obtained with varying initial conditions for cell *A*, as explained subsequently. To ease the computational analysis, we work in scaled dimensionless units. We choose cell size (diameter) σ (see Figure 1), diffusion time $\sigma^{2}/D_{0}$, and thermal energy $k_{B}T$—with *T* corresponding to the temperature of the cell/substrate system—as our length, time, and energy scales, respectively. Equations (1)–(3) may then be rewritten as

(4) $$\frac{dr^{*}}{dt^{*}}=-\frac{dW^{*}}{dr^{*}}+\sqrt{2D_{T}}\;\;\;\eta_{T}^{*},$$

where the potential in scaled form is

(5) $$\begin{array}{ccc} W^{*} & = & \frac{1}{2}k_{\mathrm{steric}}{\left(1-r^{*}\right)}^{2},\;\;\;\mathrm{when}\;\;\;0\leq r^{*}\leq 1,\;\;\;\mathrm{and} \end{array}$$

(6) $$\begin{array}{ccc} & = & -\frac{\alpha }{r^{*3}},\;\;\;\;\;\mathrm{when}\;\;\;r^{*}>1. \end{array}$$

The superscripts ${}^{*}$ in Equations (4)–(6) denote non-dimensional quantities. Henceforth, we will drop this subscript for clarity. Thus, the dynamics may be followed as a function of three dimensionless numbers (parameters)

(7) $$\alpha \equiv \left(\frac{P^{2}\phi \left(\nu \right)}{Ek_{B}T\sigma^{3}}\right),\;\;\;\;D_{T}\equiv \left(\frac{D_{\mathrm{eff}}}{D_{0}}\right),\;\;\;\;\;\mathrm{and}\;\;\;\;k_{\mathrm{steric}}\equiv \left(\frac{k\sigma^{2}}{k_{B}T}\right).$$

### 3.2. Dimensionless Parameters Quantifying Cell Motion and Interactions

Table 1 summarizes the parameters that emerge in Equations (1)–(7) and that are typical of the two-cell scenario studied here. Following Ref. [3], we are interested in substrates that are linearly elastic with the Young’s modulus *E* ranging from 0.5 kPa to 33 kPa, well within the range of 0.1–100 kPa appropriate for tissues and bio-compatible materials [5]. The effective diffusion coefficients that are exhibited by cells in the experiments [3] include the random noisy motion as the cells explore territory and a contribution due to short-time deterministic motion. We explore values in the range of 3 μm^2^/minute to 50 μm^2^/minute. Time scales are estimated from experiments as well and 250 s in real time correspond to a dimensionless time duration of unity.

Scaled non-dimensional parameters that are relevant to the simulation may be calculated from dimensional quantities, as explained earlier. Three scaled parameters determine the dynamics of the two-cell system: $D_{T}$, α, and $k_{\mathrm{steric}}$. Table 2 lists the values used in the computations. The self avoidance parameter $k_{\mathrm{steric}}$ is chosen, such that the cells do not overlap, and it is computed based on the time step used in the simulations. This allows us to control the stability of the simulation and its accuracy.

### 3.3. Numerical Solution and Tracking Cell Trajectories

Equations (4)–(7) are solved for the dynamics of the moving cell with appropriate boundary and initial conditions. The Langevin Equation (4) is an example of stochastic differential equations; here, we solve this equation using the explicit half-order Euler–Maruyama method that one of us has used recently in similar problems involving bacteria cells moving in light fields [42] and in simulations of active Brownian particles [48]. Given the position of cell A at time *t*, r(t), its subsequent location at time t+δt, r(t+δt), follows,

(8) $$r\left(t+\delta t\right)=r\left(t\right)-\left(\frac{\partial W}{\partial r}{|}_{r(t)}\right)\delta t+\sqrt{2D_{T}\delta t}\;\;w,$$

In Equation (8), w is a random two-dimensional vector with components each being drawn at every time step from a normal distribution with mean zero and standard deviation of unity. We simulated several trajectories of cell A (N=1000 trajectories, cells have diameter σ=1 in scaled units), under the influence of the central stationary cell B (also having diameter σ=1). The simulations were conducted in two different geometries, as described below. To study the contact frequency between two-cells and systematically explore the role of the elastic potential, we simulated cell A moving in a confined square box of size 12σ with the stationary cell B at the center of the box. The cells reflect from the box surface when they encounter it and, thus, are restricted to remain within the simulation domain.

We define a contact radius 1.5σ from the centre of the stationary cell, and we consider a contact if the centre of the test cell lies within the contact radius, in order to calculate the number of contact in due course of the simulation. The cell can come out of the contact radius and re-enter, increasing the number of contacts. The time step used in these simulations is dt=0.0001 and the total number of steps in this simulation is $10^{7}$, i.e., a cell trajectories were followed for a total time of T=1000.

On the other hand for calculating cell dispersivities, and specifically the mean squared displacement (MSD) of cell A, we used periodic boundary conditions and a periodic potential. This corresponds to cell A moving in a periodic domain and interacting with a regular square lattice of multiple stationary cells (images of B) separated uniformly by a distance 12σ. The time step that is used to integrate Equation (7) in these simulations is also dt=0.0001 and the total number of steps in this simulation is $10^{7}$, i.e., cell trajectories were followed for a total time of T=1000. The mean square displacement MSD was calculated by tracking trajectories of cell A (the same as tracking N=100 cells). As before, cell A is randomly initialized inside the same square box of length of 12σ, but outside the contact radius. Cells that move out of the domain are reintroduced into the domain in a manner that respects periodic boundary conditions and the appropriate symmetries.

In this case, since $r\equiv xe_{x}+ye_{y}$ is the relative distance between the cells, the mean square displacement is calculated by the equation,

(9) $$\mathrm{MSD}\left(\tau \right)=\frac{1}{N}\sum_{\alpha =1}^{N}\langle {\left[x_{\alpha }\left(t_{R}+\tau \right)-x_{\alpha }\left(t_{R}\right)\right]}^{2}+{\left[y_{\alpha }\left(t_{R}+\tau \right)-y_{\alpha }\left(t_{R}\right)\right]}^{2}\rangle$$

where τ is the delay time, and the summation is over each cell trajectory (indexed by α) and it extends over the full number of trajectories N=100. The delay time is varied and the averages are obtained by choosing different values of the reference time $t_{R}$, as is normally done.

Thus, the MSD is an average over time and also an average over realized cell trajectories. We also note that the mean square displacement in (9) is written as a function of the delay time τ that may be interpreted as an effective observation time over which the cell motion is observed.

The mobility of cell A reflects the properties of the microenvironment created by cell B and the substrate. For instance, a cell that moves with constant speed for small times (say $∼T_{1})$ and undergoes a diffusive random walk when observed over long times (say ∼$T_{2}$) will exhibit different slopes for $\tau <T_{1}$ and for $\tau >T_{2}$. The exponent characterizing the dependence of the MSD on the delay time provides information regarding whether the motion is sub-diffusive (exponent <1), diffusive (exponent =1), or super-diffusive (exponent >1).

It is constructive to study the expected MSD for cell A in the absence of cell B. In this particular case, since A is purely diffusive, the MSD has the simple form that is valid for diffusion in two dimensions $\mathrm{MSD}\left(\tau \right)=4D_{T}\tau$. Deviations from this expression arise due to the mechanically induced inter-cell interaction and, thus, quantify the extent to which cell B perturbs the dispersion of cell A. For instance, transient or persistent trapping of cell A will result in the MSD scaling sub-linearly with τ.

## 4. Results

### 4.1. Cell–Cell Contact Frequency Is Controlled by Matrix Elastic Interactions

Being motivated by experiments that show that two cells make repeated contact and withdrawals on soft substrates, with contact frequency dependent on the substrate stiffness, we measure the total number of contacts of the motile cell (A) with the stationary cell (B) in our model simulations. As indicated earlier, the simulated cells are initialized randomly inside the box, but outside of a pre-defined contact radius around the stationary cell. The total number of contacts between the cells is counted over a fixed period of time i.e., T = 1000. It should be remembered that the cells are confined to stay within the square domain during the course of the simulation.

Cell A’s movement is governed by an attractive elastic potential that is induced by the stationary, central cell, and its own random motion, described as an effective diffusion. Additionally, when the cell encounters the bounding wall of the square domain, it reflects (moves away) from it. Overall, random noise encapsulated in the diffusion coefficient causes A to move towards or away from B in an unbiased manner. The attractive potential *W* being isotropic and spatially varying suggests that there is a critical radius of influence (dependent on both α and $D_{T}$), within which forces due to the attractive potential dominate diffusion and significantly influence the trajectory of cell A. This effect results in the cell getting closer to cell B, eventually entering this zone of influence.

**Figure 3:**
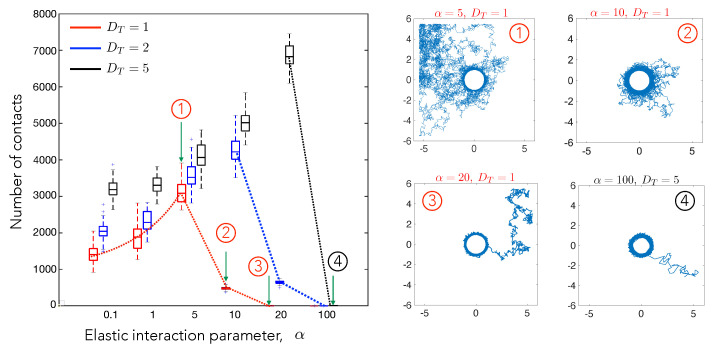
The number of cell–cell contact events measured in a fixed interval of time depends strongly on the elastic interaction parameter. A contact event is identified as cell A coming within a prescribed contact radius of cell B with cell A initialized randomly in a certain area around cell B. Thus the number of contact is be interpreted as the average number of contacts of the two cells. The number of simulation runs conducted were 50 for each combination of $D_{T}$ and α. The dashed curves are guides to the eye illustrating the trends seen with increasing values of α. Diffusion is the major factor in governing the number of contacts for low values of α. For higher α, the attractive potential increases the probability of the cell to stay near the contact radius and controls the number of contacts. The trajectories for highlighted data points (1)–(4) are shown on the right. The box plots show the distribution of contact numbers. The lower and upper bounds of the box are the first and the third quartiles respectively, while the line in middle is the median. The lower and upper limits of the dashed lines are the minimum and maximum number of contacts observed for cells for each combination of α and $D_{T}$. The simulation was run for a total time of T=1000 and updates in the cell position were made every δt=0.001.

To carefully study how elastic interactions (α) and random diffusion ($D_{T}$) each influence this process, we first systematically calculated the number of contacts by α, while keeping $D_{T}$ constant at three different values, $D_{T}=1,2,5$. (Figure 3). The behavior is highly non-monotonic, as illustrated by the dotted lines which serve as a guide to the eye. For small α, the number of contacts increases with increasing α, then reduces to 1 at high α. The position of the peak increases with increasing $D_{T}$. The initial increase in contacts is due to the increased directional movement of the test cells towards the central cell. The decrease in the number of contacts for very high values of α is expected, since the attractive potential is strong enough to overcome the effect of diffusion. In this case, the motile cell is unable to move away from and it makes stable contact with the stationary cell. For α=5 and $D_{T}=1$ (trajectory 1), the test cell spends most of the time exploring space rather than near the stationary cell, which also reduces the number of contacts.

Upon increasing α to 10 (trajectory 2), the radius of influence increases, increasing the duration of contact, and thereby increasing the contacts. On further increasing α to 20 (trajectory 3), the test cell is tightly adhered to the stationary cell which allows only one single contact. Note that the statistics for the high $D_{T}$ and low α regime are influenced by the confinement. Cells in this particular limit frequently escape the region of influence and wander away, only to return again after encountering the wall and diffusing away. For instance, the number of contacts for $D_{T}=5$ and α=0.1 combines the effect of repeated escapes from the region of influence and repeated returns due to confinement. Because the size of the box is fixed, the increase in number of contacts with $D_{T}$ for α=0.1 is still a signature of diffusive effects dominating the attractive potential.

**Figure 4:**
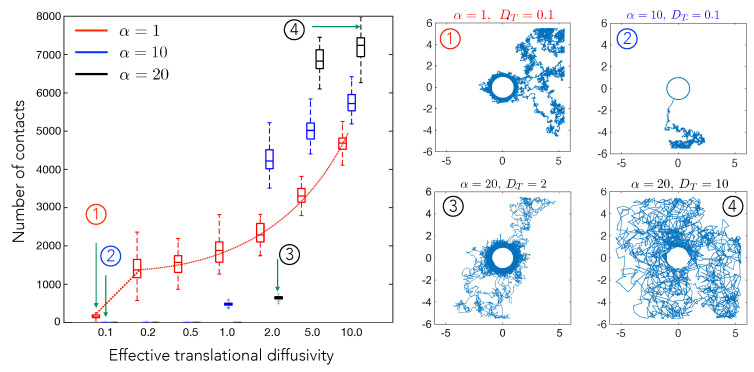
The number of cell–cell contact events in a fixed interval of time (T=1000) plotted here as a function of the scaled effective diffusivity, $D_{T}$, which represents the random motility of cell B. Here, we show how the number of cell–cell contact varies for three different elastic interaction strength values, α, corresponding to substrates with three different stiffness. The highlighted points numbered from (1)–(4), show representative cell trajectories over long times and highlight how varying α and $D_{T}$ can yield states where the cells are in close proximity most of the time (low $D_{T}$, high α) or states where cells interact rarely (high $D_{T}$, low α). The interpretation of the box plots is the same as in Figure 2. The simulation was run for a total time of T=1000 and updates in cell position were made every δt=0.001.

We next investigated the effect of increasing diffusivity on the number of contacts for constant α (1, 10, and 20). The results from this set of simulations are shown in Figure 4. The red dotted line serves as a guide to the eye, highlighting the trend observed. We see a steady increase in cell–cell contacts with diffusivity. Without diffusion, the test cell shows unidirectional motion towards the central cell and remains in contact throughout the simulation. Increasing diffusion increases the chance of test cell to go out of the radius of influence and then come back again (trajectories 3 and 4).

Overall, combining the results that are shown in Figure 3 and Figure 4, we conclude that the number of contacts is maximized at an optimal value of the elastic interaction strength. If the elastic strength is too high or too low, the cell either makes stable contact or is too motile to make too many contacts. This optimal value scales with the diffusivity, which is a measure of the cell motility in our model. We note that these are precisely the behaviors seen in experiments on two-cell interactions and motility, suggesting that purely long-ranged mechanical cell–cell interactions suffice to predict the contact frequency and the effective duration for which two cells can remain close to each other. This is important, because, for short-range interactions and specific biochemical recognition mechanisms to be initiated, cells have to first be drawn together. Our results suggest that mechanical interactions may play an important role in first getting cells close to one another in order for subsequent attractive or repulsive cell-cell direct interactions to then turn or and control subsequent dynamics. Taken together, our simulations suggest that elastic interactions can lead to stable contact between initially distant cells.

### 4.2. Cell Motility Characteristics Depend on Elastic Interactions

To quantify the long-time statistics of the motility of cell A in the elastic potential field that is generated by cell B, we analyze the mean squared displacement (MSD), as given by Equation (9) from simulation. The metric MSD measured in terms of a delay time τ contains information regarding the short time mobility of a cell, the long time mobility of the cell, and additionally provides signatures of capture and trapping effects. Specifically, the slope of the mean square displacement can be used to extract effective exponents that provides insight on the relative importance of diffusion and elastic attractive interactions.

**Figure 5:**
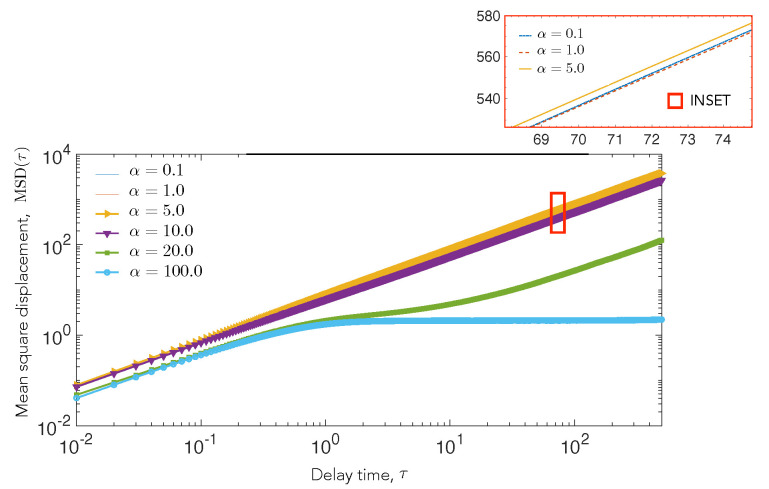
Mean square displacement (MSD) as a function of the delay time interval τ (calculated from Equation (9)), for the motile cell A is shown. Here we explore the variation in the MSD for various values of substrate-mediated elastic interactions, α. The diffusivity $D_{T}$ is held constant for these simulations with $D_{T}=2$. Other diffusivities were explored (results not shown). At low elastic interaction strengths, α, corresponding to stiff substrates, the cell shows a purely diffusive trajectory, whereas at higher values of α, the motile cell is captured by the strong attractive interaction from the stationary cell, resulting in a flattening of the MSD (blue curve). At an intermediate interaction regime (green curve), the motile cell makes repeated contact with the fixed cell, but it is never fully captured.

We plot the MSD in Figure 5 for $D_{T}=2$ and α=0.1,1,5,10,20,100. For α=0.1,1,5,10, we find that the slope is close to 1, which suggests that diffusion drives the motion of the cell and the attractive potential is not strong enough to influence the movement of the cell. For higher α, we observe a transition towards sub-diffusive behavior at τ∼0.5. At α=20 (green line), the curve shows a significant decrease in slope at τ=2, the time scale for which a test cell, on average, encounters the central cell for the first time and stays in contact for a while, as shown by trajectory 3, Figure 4. The slope then increases again, but it remains less than 1, suggesting a sub-diffusive behavior in the long run. At α=100 (blue line), the MSD saturates after initial diffusion to a zero slope, which suggests that the motion is bounded, and it can only explore the circumference of the stationary cell.

### 4.3. Elastic Interactions Lead to Effective Capture of Motile Cell

Figure 3, Figure 4 and Figure 5 suggest that the motile cell A (as it explores space and samples the potential field over its various trajectories) is attracted to the stationary cell with the attracting force increasing with decreasing distance *r*. Acting in tandem and superposed on this aspect of the motion is diffusion that allows A to wander away from B multiple times. Therefore, we next explore the statistics of this capture process. Capture mechanisms underlying and influencing these statistics are potentially relevant for timescales of contact formation between initially well-separated motile cells that then form confluent monolayers, such as in mesenchymal-to-epithelial transitions during tissue morphogenesis [54].

**Figure 6:**
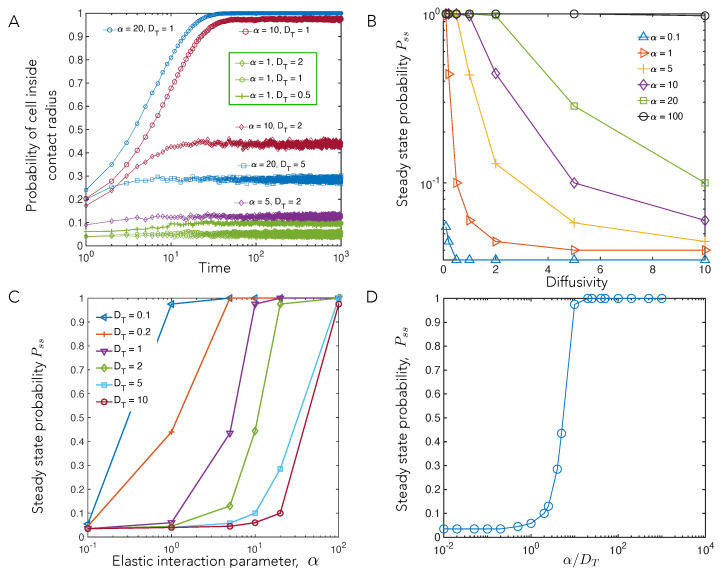
Capture statistics of motile cell. (**A**) Probability that cell B is inside contact radius as a function of time. (**B**,**C**) The dependence of steady state capture probability, $P_{ss}$, i.e., the fraction of cells captured within the contact radius after a long time interval, on simulation parameters. (**B**) shows the dependence on diffusivity, $D_{T}$ at different values of the elastic interaction parameter, α, whereas (**C**) shows the dependence on α for different values of $D_{T}$. (**D**) The steady state capture probability, $P_{ss}$, data can be collapsed into a single master curve, when plotted vs. the key parameter, $alpha/D_{T}$, the strength of the elastic interactions relative to the diffusivity. This is expected since our model steady state is a thermal equilibrium with the effective temperature set by the noisy cell motility, $D_{T}$, and the competition between attractive interactions and noise dictates the number of cells (cell trajectories) captured vs. the number that escape.

We tracked the number of cells inside the contact radius over the course of the simulation in order to understand how parameters α and $D_{T}$ affect this phenomenon. The probability of cells inside the contact radius reached a steady state at time t<100 for all parameters (Figure 6A). Keeping α constant and increasing $D_{T}$, the probability of cells being inside the contact radius decreases (Figure 6B). The steady-state probability $P_{ss}$ increases with an increase in α for constant $D_{T}$ (Figure 6C). To understand the relationship between $P_{ss}$ and both α and $D_{T}$, we investigated $P_{ss}$ for the ratio $\alpha /D_{T}$, and showed that they remain constant for this ratio.

Plotting $P_{ss}$ vs $\alpha /D_{T}$, the strength of the elastic interactions relative to the diffusivity, we find that the data can be collapsed into a single master curve (Figure 6D). The collapse of our data and the master curve plotted in Figure 6D is expected; meanwhile, the competition between attractive interactions and noise dictates the relative numbers of cells that are captured vs. cells that escape.

Figure 6 motivates thinking about the moving cell as exploring a special region where substrate mediated elastic cell–cell interactions dominate. This region has a characteristic radius of influence equal to the distance from the stationary cell at which its elastic attractive tendency approximately balances the random noisy movements of the motile cell. Here, we use a simple balance to estimate this radius of influence. Working in dimensionless units, we note that the dipolar interaction potential fall off as $\alpha /r^{3}$, while the effects of the randomizing diffusion scale as $k_{B}T=\mu_{T}D_{T}$. Balancing these yields, a length scale (for mechanical interaction) $\ell_{M}∼{\left(\alpha /\mu_{T}D_{T}\right)}^{1/3}$, which explicitly shows the importance of the $\alpha /D_{T}$ parameter. Thus, a stronger α from deformations exerted by the stationary cell (corresponding to softer substrate stiffness, or higher contractility) and lower random movements of the motile cell, $D_{T}$, leads to a larger radius of influence. This, in turn, implies that the probability of being captured within the contact radius increases because the stationary cell can influence motile cells over a larger area.

## 5. Discussion and Future Extensions to Other Forms of Interactions

### 5.1. Anisotropic Cell-Cell Elastic Interactions

For polarized cells, which orient their cytoskeletal fibers and contractility along some principal axis, the cell–cell interaction potential is not isotropic. The individual cells on an elastic medium behave as force dipoles, with the interaction potential energy having both attractive and repulsive regions that depend on the mutual orientation of the two cells and their separation vector [39], as detailed in Appendix A. The force experienced by the motile cell has both radial and tangential components, depending on its position and orientation relative to the central cell, and its direction is sensitive to the Poisson’s ratio of the elastic medium [55]. Thus, trajectories of cell A interacting with stationary cell B when the fully anisotropic interaction potential (Equations (A1) and (A2), Appendix A) is included will differ from the trajectories observed in isotropic potentials. The difference arises in part due to an additional torque that reorients cell A to preferentially align with cell B as it moves towards it. Nonetheless, the qualitative nature of the capture process and the observation of an effective region of influence will still remain valid.

Here, we would like to point out that the term anisotropy of the interactions is coupled to the directionality of the dipole axis quantifying the contractile stresses that are exerted by the focal adhesions on the underlying substrate. The substrate by itself is still treated as a homogeneous linearly elastic isotropic material; the anisotropy specifically refers to the fact that cell-cell interactions are not not just dependent on the inter-cell distance, but also on the relative angles between their dipole axes. This is important, for instance, in cells that vary their direction of motion slowly, or equivalently bleb, and change their direction of motion very slowly. In this scenario, the direction each cell is moving along is as important as the inter-cell distance (see Appendix A).

**Figure 7:**
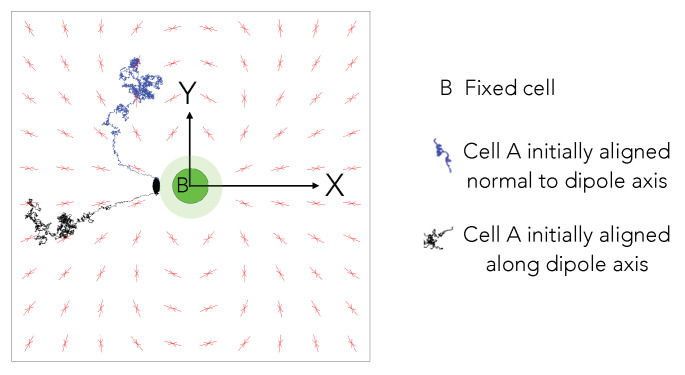
Dipolar cell orientation and trajectory. The equilibrium orientation of contractile cells fixed in position, but free to reorient, and that are uniformly distributed in a square box of size 10σ, are depicted by two arrows (red) pointing towards each other. Each cell is influenced by the central stationary cell B (green) and not by each other. Two possible trajectories of cell A (blue and black) are recorded for $D_{T}=0.1,\;\alpha =40$ for total time T=500 with time steps of dt=0.001. The cells did not have any self propulsion or rotational diffusion. The Poisson’s ratio ν of the substrate was considered to be 0.3 for this simulation.

To illustrate this, we simulated the equilibrium orientation of uniformly spaced (pinned) test dipolar cells on a square lattice that are kept fixed in a square box of length 10σ. The Poisson’s ratio of the simulated substrate is 0.3 and α is 40. Figure 7 shows the results. None of the cells overlap with the central stationary cell; they may rotate to reorient their dipole axis but are restricted from translating. We re-iterate that the cells on the lattice do not mutually interact with each other, but they are only meant to illustrate the interaction of the test dipolar cell A placed at different spatial locations with the central stationary cell B. We note that fixed cells adjust the axis of their contractile dipoles in accordance to the potential field due to cell B (the dipole axis of B is fixed).

Superposed on this are two trajectories that correspond to two cells that are freed from constraints and allowed to rotate and translate in response to the two-cell potential and thermal noise. The two cells start from their equilibrium orientation—i.e, they are first held pinned and allowed to reorient until the dipole axis attains a static value and then the pinning constraint is removed. The cells in close vicinity of the central cell’s orientation axis exhibit a nearly linear motion to the pole of the fixed cell (trajectory in black). Cells away from the orientation axis take a longer route to come in contact with the central cell (trajectory in blue). The common attribute in both trajectories is that they prefer to adhere to the central cell’s pole, which is cell A, as it moves towards B, also continuously reorients in a manner that brings it into alignment with the cell B’s polar axis (the axis of the dipole).

### 5.2. Extensions to Near-Contact Biochemical or Bond Interactions

In many realistic biological settings, several different modalities mediate cell–cell interactions, sometimes with parallel intercellular signaling events occurring together. The interplay between these different modalities is still unexplored. In addition to long range mechanical interactions, cells may sense other cells by short-range mechanosensation and adhesion mechanisms, including ligand based bond interactions, by biochemical signalling and other molecular recognition mechanisms [22], by haptotaxis [23], as well as by responses to specific molecules or signals put forth by neighboring cells [25,27]. A salient example is that of immune cells that can sense and respond to biophysical cues—from dynamic forces to spatial features—during their development, activation, differentiation, and expansion, as well as to biochemical cues. The biophysical signals modulate immune cell functions, including leukocyte extravasation, macrophage polarization, T cell selection, and T cell activation [56,57]. Meanwhile, cell–cell interaction between cells in close proximity may be impacted strongly by biochemical interactions. In lymphocytes, T cell receptors and B cell receptors recognize antigens and activate effector functions that combine chemical and mechanical features. In general, near-contact encounters between cells may be positive (attracting), as between T lymphocytes and B cells, or negative (repulsion), as occurs between IL-1-producing macrophages and endothelial or immune cells.

The agent that is based the Langevin equation model here provides a foundation to analyze some forms of these biochemical or near-contact interaction mechanisms. As a first example, we consider integrin–ligand interactions that are hypothesized to mimic catch bonds [58,59,60,61]. In previous work, one of the authors analyzed the kinetically driven capture of a cell (such as a leukocyte) by binders that were attached to a stationary surface [62]. In the context of the two interacting cells discussed here, similar near-contact binding interactions can be simulated by positing a distribution of bonds (with attachment and detachment probabilities/rates drawn from detailed mechanochemistry) on the surface of each cell. If mechanochemistry dictates that binders interact when the inter-cell distance is $\ell_{B}$, then, for soft to moderate substrates, we may expect $\ell_{B}\ll \ell_{M}$. That is, mechanical interactons are crucial in enabling close contact between cells first before the binder interactions kick in and either capture (attract) or deflect (repulse) the cell. A detailed model for this process is a part of current work.

As a second example, we consider motile cells that are elastically coupled, and biochemically coupled through chemical agents and matching receptors. A specific example related to cultured microglia—the immune cells of the brain—where a number of chemical markers (e.g., α5-integrin) are left behind on the substrates as cells move with these surface bound degradable markers serving as signaling agents guiding two-cell interaction [23,24,25,26,27,28]. This process by which microglia exhibit haptotaxis by following these chemical signal trails they generate may also be studied by coupling Equations (1)–(7) or, more generally, Equations (A1)–(A6) to a scalar equation for the time-dependent concentration field of these chemical markers. The limiting case where mechanical interactions are absent has been addressed earlier (see, for instance, [23]).

Finally, the computational framework introduced and analyzed here can be extended to study durotaxis—that is, the modification of cell motility by variations in substrate elasticity at the single cell or tissue level and the motion of cells towards higher stiffness regions [63,64].

## 6. Summary

Using our model for cell contractility and motility, we computed several metrics of experimental relevance, such as number of cell–cell contacts, the mean square displacement of a motile cell in the presence of elastic deformations induced by a cell in its vicinity, and associated capture statistics that result from attractive interactions between two such cells. In each case, we predict how the computed metric depends on the elastic properties of the substrate, captured in the interaction parameter, α∼1/E, and on cell motility, captured by the effective diffusivity, $D_{T}$. Our results support the hypothesis that cell–cell interactions with purely mechanical origins can lead to mutual contact without requiring specific chemical factors to guide their motility. Our model also predicts that substrate stiffness is an important control parameter in guiding cell motility and forming multi-cellular structures.

Similar to the observations for pairs of endothelial cells mechanically interacting through the compliant substrates [3], we find that the motility and number of cell–cell contacts are lowered at large α, corresponding to softer substrates. This is because the elastic deformations of the substrate and, therefore, the cell–cell attractive interactions are stronger when compared to the random motility. As observed in the experiments, we also find that, at intermediate interaction strength, the cells can make repeated contacts and withdrawals, as shown in the contact number measurements. For very stiff substrates, which is low interaction strength, we find the cell remains diffusive and can migrate away from the stationary cell and does not make frequent contacts. Our findings would therefore suggest an optimal substrate stiffness at which contact frequency is maximal. These trends are also reflected in the MSD measurements. Unlike the experiment, we do not find diffusive MSD for the strongly attractive case, but the MSD turns sub-diffusive, which suggests perhaps that such high interaction strengths were not probed in the experiment.

Biologically, altered motility and contact formation can be relevant for forming stable adhesive contacts between cells and tissue development, including that of blood vessels during vasculogenesis [65]. In our approach and in formulating the minimal model, we made several simplifying assumptions in the model (as stated in Section 2), including using a purely attractive and isotropic potential instead of the dipolar potential that is relevant for elongated and motile cells. Figure 6 illustrates how the position and orientation of the motile cell with respect to the stationary cell leads to qualitatively different trajectories when the interaction potential is dipolar. Such an anisotropic potential is expected to lead to end-to-end alignment and contact formation of a pair of cells. With multiple cells, larger scale structures, such as chains and networks of cells, can result [39]. The influence of cellular motility on these structures will be the topic of a future study.

Cells are typically soft with values of estimated elastic moduli that are approximately equal to the range that we have used here for the substrate stiffness. One may ask how the nominal cell stiffness or elasticity enters the model and the range of validity of the model. We note that our model, by itself, holds for stiffer substrates as far as the response is that of a linear elastic material. With regard to the manner by which cell properties are implemented in the agent-based model, we point out that the underlying theoretical model that forms the basis of our agent based simulations treats the cell as a line dipole exerting contractile stresses on the substrate. In this minimal framework, properties of the cell, such as its stiffness and the variation of the contractile stress with both cell properties (such as type and stiffness), are encapsulated in the dipole strength.

To improve on this further, we briefly consider the recent advances that shed light on how cell (cortical) stiffness, the mechanical properties of the focal adhesion regions, and alignment of intracellular structures affect the dipole strength. Recent work paints a complex and tightly coupled picture. (a) Cell elastic modulus is not a constant, but dynamically and continuously altered dependent on substrate stiffness and topography (see [66], for instance). (b) Thus, the coupling between surface stresses, substrate stiffness, and focal adhesion dynamics is emergent, and it cannot be predicted *apriori*. It was recently shown in experiments using mouse embryonic fibroblasts [67] that vinculin regulates force transmission to the substrate and vinculin and focal adhesion area did not correlate with traction force magnitudes at single focal adhesions, and this was consistent across different ECM stiffness and cytoskeletal tension states. Vinculin residence time varied linearly with applied force for stiff substrates; this was disrupted on soft substrates. (c) There is strong indication that local stiffness and the alignment of intrafiber aggregates in the cell–substrate contact area are important. Vargas et. al [68] hypothesize that the point at which substrate stiffness becomes as high as that of the cell–cell interface corresponds to configurations where cell–substrate biophysics may dominate. (d) Atomic force microscopy (AFM) indentation measurements of cells that were cultured on deformable substrates suggest that cells adapt their stiffness to that of their surroundings. Recent work [16] probed the elasticity of microglial cells and fibroblasts that were cultured on soft matrices mimicking the mechanical properties of the physiological cell environment. When combining analytical estimates from Hertz contact theory with detailed finite element modeling, they found that cell stiffness was rather insensitive to substrate stiffness. These studies indicate that significant work still remains to be done in order to clarify the roles that are played by cell stiffness and substrate stiffness.

In this paper, we used a minimal model, where the microscopic details are coarse-grained. More detailed models and descriptions of cell and substrate deformation can be included if needed using finite element methods coupled with mechanochemical models that take detailed deformation history of the substrate and the cell–substrate region into account. The advantages of complementing experimental studies with modeling approaches as discussed in this paper is that hard to realize parameter regimes may be easily investigated. Furthermore, the role of different physical parameters may be clearly studied in isolation; a feature that is hard to achieve in an experimental setting. Understanding the mechanistic aspects of cell–cell interactions, as done here, has implications for regenerative medicine and tissue engineering, and it will guide and inform experiments exploring how cells communicate with each other in the process of organizing and moving collectively.

## Figures and Tables

**Table 1:** The biophysical parameters characterizing the two-cell (typical values from [3,52,53]).

Quantity	Interpretation	Experimental Values
σ	Cell size	10–100 μm
*T*	Temperature	25 °C
$D_{0}$	Thermal Diffusivity	25 μm^2^/min
$D_{\mathrm{eff}}$	Effective Diffusivity	3–50 μm^2^/min
*E*	Young’s modulus	0.5–33 kPa
ν	Poisson ratio	0.3–0.5
*P*	Contractility	$10^{-14}$ Nm

**Table 2:** Simulation parameters and their meaning.

Parameter	Interpretation	Definition	Simulation Values
$D_{T}$	Diffusivity	$D_{\mathrm{eff}}/D_{0}$	0.1–10
α	Cell-cell interaction	$P^{2}\phi \left(\nu \right)/\left(Ek_{B}T\sigma^{3}\right)$	0.1–100
$k_{\mathrm{steric}}$	Self-avoidance	$k\sigma^{2}/k_{B}T$	$10^{3}$–$10^{4}$

## Data Availability

Data is contained within the article.

## Abbreviations

The following abbreviations are used in this manuscript:

MSD	Mean Square Displacement

## Appendix A Model for a Moving Cell Interacting with a Stationary Cell Via Substrate Elasticity

The flat substrate is treated as being semi-infinite (Figure 1) and comprised of a linearly elastic, isotropic gel-like material with Young’s modulus *E* and Poisson’s ratio ν, that capture its stiffness and compressibility respectively. The minimal model that describes the deformations created by cells exerting contractile forces on the substrate is a point-like force dipole [52]. Two identical dipolar cells denoted by *A* and *B* move in the upper plane (chosen to be the x-y plane, see Figure 1). Cell *A* is allowed to move and its dynamics is specified completely by its location on the substrate $r^{A}\left(t\right)$ and by its self-propulsion direction $e^{A}\left(t\right)$. Cell *B* is held fixed at point $r^{B}$. As a result of the contractile dipoles exerted on the substrate the cells communicate elastically. The potential $W^{AB}$ characterizing this elastic interaction between the two cells is given by

(A1) $$W^{AB}\left(r\right)=P^{2}e_{j}^{B}e_{i}^{B}\partial_{j}\partial_{l}G_{ik}^{AB}\left(r\right)e_{k}^{A}e_{l}^{A},\;\;\;\;\mathrm{with}\;\;\;\;r=r^{A}-r^{B},$$

where *P* is the strength of the force dipole capturing the contractile stresses exerted by a cell on the medium. In writing (A1), we have made the plausible assumption that cells orient their cytoskeletal structures such as stress fibers and exert their traction primarily along their motility axis, such that the force dipole tensor, which captures the moment of their force distribution, is assumed to be, $P_{ij}=Pe_{i}e_{j}$. The tensor

(A2) $$G_{ij}^{AB}\left(r\right)=\frac{1+\nu }{\pi E}\left[\left(1-\nu \right)\frac{\delta_{ij}}{r}+\nu \frac{r_{i}r_{j}}{r^{3}}\right],$$

is the Green’s function that captures the displacement in the elastic medium at the location of one cell (dipole) caused by the application of a point force at the location of the other [69]. The partial derivatives in (A1) on the right hand side are taken with respect to relative position vector r. Standard Einstein notation has been chosen in writing the form of $W^{AB}$ and the derivatives in Equations (A1) and (A2).

To obtain the force and torque balance equations that govern the dynamics of cell *A*, we make the simplifying assumption that the cells move in an overdamped fashion. This implies that hydrodynamic interactions between cells are ignored, and that each cell feels a resisting viscous frictional drag/torque that is proportional to its velocity/rotation rate. Conversely, when acted on by a force F or a torque T, a cell in this overdamped environment will move with velocity $\mu_{T}F$ or rotate at a rate $\mu_{R}T$ respectively. Here, $\mu_{T}$ and $\mu_{R}$ are appropriate mobility terms that depend on the cell size.

The micro-dynamics of cell *A* moving on the substrate is governed by the Langevin equations for the translation and rotary motion of cell. Recognizing that the elastic interaction generates (extra) forces and torques that act on each cell, and including the effects of fluctuating time dependent forces $\xi^{T}\left(t\right)$ and torques $\xi^{R}\left(t\right)$ originating from thermal noise, we can write the equations for the position and orientation of cell *A* in the presence of cell *B* as

(A3) $$\begin{array}{ccc} \frac{\partial r^{A}}{\partial t} & = & v_{0}e^{A}-\mu_{T}\frac{\partial W^{AB}}{\partial r^{A}}+\mu_{T}\xi^{T}\left(t\right),\;\;\;\;\mathrm{and} \end{array}$$

(A4) $$\begin{array}{ccc} \frac{\partial e^{A}}{\partial t} & = & -\mu_{R}\left(e^{A}\times \frac{\partial W^{AB}}{\partial e^{A}}\right)+\mu_{R}\xi^{R}\left(t\right). \end{array}$$

In an equilibrium situation, the random forces and torques are white noise terms and are related to one another by the equipartition and fluctuation-dissipation theorems: $\langle \xi^{T}\left(t\right)\xi^{T}\left(t^{\prime }\right)\rangle =\left(2k_{B}T/\mu_{T}\right)\delta \delta \left(t-t^{\prime }\right)$ where δ is the Kronecker delta function. For active cells however, these restrictions do not hold; these terms are set by active internal cell responses to the substrate properties. Equations (A1)–(A4) are used in the results illustrated in Figure 5.

In the bulk of the paper and for results presented in Figure 1, Figure 2, Figure 3 and Figure 4, we use an isotropic version of the potential in Equation (A1) that ignores orientational dynamics that are in general present for highly elongated cells. This assumes a separation of scales between the time over which cells reorient and the dipole axis changes and the time for the center of the cell to move significantly such as when the rotation noise in (A4) is significant. In this limit, one can average over the rapid reorientations of the cells and replace $e_{j}^{B}e_{i}^{B}$ by $\delta_{ij}$ and $e_{k}^{A}e_{l}^{A}$ by $\delta_{kl}$. Equation (A1) then reduces to the simpler form that we employ in the main discussion of the paper and implement as a numerical simulation,

(A5) $$W^{AB}\left(r\right)=P^{2}\partial_{i}\partial_{k}G_{ik}^{AB}\left(r\right)=\frac{P^{2}}{E}\frac{\phi (\nu )}{r^{3}}$$

with the function $\phi \left(\nu \right)=\left(1-\nu^{2}\right)/\pi$ dependent solely on the Poisson ratio, and hence fixed in the simulation. Furthermore, since the dipole axis of cell *A* reorients in time scales much faster than its slower rate of translation, the $v_{o}e^{A}$ term in (A3) simplifies to a time fluctuating variable with a mean that is roughly zero but with a non-zero variance. Thus its net effect may be incorporated by appropriately modifying the translational diffusivity. For an isotropic symmetric potential as here, the equation that needs to be solved is then

(A6) $$\frac{\partial r}{\partial t}=-\mu_{T}\frac{\partial W^{AB}}{\partial r}+\mu_{T}\xi_{*}^{T}\left(t\right),$$

with the modified random force $\xi_{*}^{T}$ reflecting an effective translational diffusivity $D_{\mathrm{eff}}$ different from the thermal diffusivity $D_{0}$, through a relation, $\langle \xi_{*}^{T}\left(t\right)\xi_{*}^{T}\left(t^{\prime }\right)\rangle =\left(D_{\mathrm{eff}}/\mu_{T}^{2}\right)\delta \delta \left(t-t^{\prime }\right)$. We define the dimensionless number $D_{T}\equiv D_{\mathrm{eff}}/D_{0}$. Consistent with this, we choose $\mu_{T}=D_{\mathrm{eff}}/k_{B}T$.
